# Challenges faced during the COVID-19 pandemic by family carers of people living with dementia towards the end of life

**DOI:** 10.1186/s12913-021-07019-6

**Published:** 2021-09-21

**Authors:** Narin Aker, Emily West, Nathan Davies, Kirsten J. Moore, Elizabeth L. Sampson, Pushpa Nair, Nuriye Kupeli

**Affiliations:** 1 Centre for Ageing Population Studies, Research Department of Primary Care and Population Health, University College London, London, UK; 2 Marie Curie Palliative Care Research Department, Division of Psychiatry, University College London, 6th Floor, Maple House, 149 Tottenham Court Road, London, W1T 7NF UK; 3 National Ageing Research Institute, Parkville, Victoria Australia; 4 Barnet, Enfield and Haringey Mental Health Liaison Service, North Middlesex University Hospital NHS Trust, London, UK

**Keywords:** COVID-19, Coronavirus, Dementia, Family carers, Concerns, Decision making, End of life, Place of care, Telephone support, Charities

## Abstract

**Background:**

People living with dementia account for a large proportion of deaths due to COVID-19. Family carers are faced with making significant and emotive decisions during the pandemic, including decisions about end of life. We aimed to explore the challenges faced by family carers of people living with dementia during the first wave of the COVID-19 pandemic in England, as reported by charity telephone support line staff, who were able to objectively discuss a range of different experiences of many different carers who call the helpline. In particular, we focussed on key concerns and areas of decision making at the end of life.

**Methods:**

We conducted a qualitative study using semi-structured interviews with eight telephone support line staff from two UK based charities who support carers of people living with dementia and those at the end of life. Interviews were conducted in the first wave of the pandemic in England in May–June 2020.

**Results:**

An overarching theme of uncertainty and reactivity during a crisis was identified, and within this, five main themes were identified: concerns about care transitions, uncertainty in engaging support and help, pandemic-motivated care planning, maintaining the wellbeing of the person living with dementia, and trust, loss of agency and confusion.

**Conclusions:**

Family carers may be reluctant to seek support because of fear of what may happen to their relative, which may include hospitalisation and becoming ill with COVID-19, care home placement, or not being able to be with a relative at the end of life. In some cases, a lack of trust has developed, and instead carers are seeking support from alternative services they trust such as nationally known charities.This study was used to inform the development of a decision aid to support family carers making decisions about care for their relative with dementia during the pandemic, who the lack the capacity to make their own decisions.

## Background

Globally, the main underlying condition for COVID-19 deaths has been dementia [1]. In the United Kingdom (UK) this has accounted for 25.6% of all COVID-19 deaths, from March to June 2020 [2].

The pandemic has resulted in a variety of new challenges and concerns for family carers in the already difficult landscape of caring for someone living with dementia (Wang et al., 2020). Health and social care services have taken time to adjust to new ways of working and some facilities, such as day centres, are still inaccessible due to COVID-19 measures. This has had an impact on carers who are supporting family members, many of whom have taken on more caring responsibilities [3]. These findings are supported by Carers UK, who found that 70% of carers are providing more care due to the COVID-19 outbreak - on average 10 extra hours per week. Over a third (35%) of carers are providing more care as a direct result of local services reducing or closing and over half of carers (55%) expressed concerns that they feel overwhelmed [4].

As dementia progresses and the person living with dementia can no longer express their wishes and preferences or have the capacity to make decisions, family members must advocate on their behalf [5]. This may involve making difficult decisions such as those concerning place of care or death. Although these types of decisions are needed to be made by families outside of the pandemic [6], the pandemic may have made these more worse.

The pandemic has had an impact on the decisions that family carers of people living with dementia have to make, including those around end of life care and place of death [7]. Decisions include where the individual should be cared for, including whether to allow home care services to continue providing care at home [8]. Family carers may need to make rapid decisions with limited support and guidance from health care professionals. For this study end of life care was not based on stage of disease, but instead considering the often prolonged and complex nature of end of life for people living with dementia, adopting a needs-based focus, as highlighted in a recent review [9].

As a result of the pandemic, the way that care is provided has changed to protect staff and patients. All staff are required to wear more extensive personal protective equipment (PPE). This can be a distressing experience for a person living with dementia, as not being able to recognise carers can cause confusion and anxiety. In the UK, visits to hospitals have been restricted so the person may be required to stay in hospital with no family to advocate for their needs, wishes and preferences for the duration of their care. It is unclear the impact this may have on carer decision making and support seeking. Where the person living with dementia is unable to communicate their needs or have a family carer advocating for their needs, health care professionals may have to rely on observable signs of COVID-19 [10], which may delay getting a diagnosis and receiving appropriate treatment. People living with dementia may present with different COVID-19 symptoms to those commonly associated with COVID-19 in the general population [11]. This could also exacerbate delay in diagnosis and create additional challenges for families; for example, hypoxia, a clinical feature of COVID-19, has been found to increase the risk of delirium in people living with dementia. This can complicate dementia presentation, cause distress for the person living with dementia, and increase care needs [12].

Guidance advises people living with dementia and their carers to engage in advance care planning in collaboration with health and social care professionals so that an individual’s preferences for end-of-life care are known [13]. The uncertainties and challenges imposed by the COVID-19 pandemic have highlighted the importance of having discussions early in the disease trajectory, to ensure the wishes and preferences of the individual are known. However, during the pandemic it has become clear many have not discussed their wishes and preferences or engaged in advance care planning. For those who did not have discussions about end-of-life care before the pandemic, advance care plans may now be influenced by the fear and emotions triggered by the pandemic rather than their actual preferences ( [14]), and many may not have the capacity to engage in discussions.

There are many changes because of COVID-19, however, there is a lack of understanding about how these changes have impacted on those living with dementia who have been adversely affect by the pandemic and those caring for them. In particular, there is a lack of information on how these changes impact decision making about care for the person living with dementia.

## Methods

### Aims

We aimed to explore the challenges faced by family carers of people living with dementia during the first wave of the COVID-19 pandemic in England. In particular, we focussed on key concerns and areas of decision making at the end of life.

### Design

Qualitative study using semi-structured online interviews, analysed using thematic analysis methods [15].

### Recruitment

Convenience sampling was used to recruit helpline support staff from two national UK charities working in dementia and end of life care. Senior management at the two organisations were approached by the research team and agreed to take part in the study. Managers sent the information sheet to all support line staff and invited staff to notify their manager if they wanted to take part. Staff were given the opportunity to speak to the research team and ask any questions ahead of interviews. The manager and research team organised the interviews at a time suitable for the participant and during a time when the support line service was adequately staffed.

### Participant demographics

We conducted interviews with eight participants in total, across two organisations. All participants had previous clinical or charity experience prior to working on the support line. Seven participants were registered nurses (with experience of working in care homes, hospital wards, community psychiatric services, social work, memory services, hospices, and palliative care), five had roles (previous or current) as nurses specialising in dementia, and one had a background in care and support work.

### Data collection

Semi-structured qualitative interviews were conducted using Microsoft Teams, a business communication platform that enables video-conferencing. Interviews were conducted through video calls with each support line worker and two researchers (interviewer and note-taker) (EW, NA), guided by a topic guide. The topic guide focused on key concerns and decisions expressed by carers of people living with dementia calling the support line during the pandemic, focussing on end of life. We probed how these concerns compared to concerns and decisions commonly reported during calls received before the pandemic from carers.

Before starting the interview, the researchers introduced themselves, explained the purpose of the interview, and informed the participant that all the information they provided would be anonymised. Participants were given the opportunity to ask any questions and have these answered before commencing the interview. Permission was sought to record each interview on Microsoft, which was granted by six participants. The interviewing researchers took detailed notes throughout the remaining two interviews. Teams, which was granted by all participants and electronic written consent was obtained via email.

Interviews took place between May and June 2020 and lasted 30–60 min.

### Data analysis

The data was analysed using thematic analysis [15]. Transcripts for each interview, created automatically by the Microsoft Teams software, were downloaded and checked against the audio recording for accuracy and uploaded to NVIVO 11 for coding. All transcripts were initially read by one researcher (NA) for familiarisation. The transcripts were coded by one researcher (NA). A second researcher (NK) read two transcripts and checked coding, the definition of codes, and a coding framework was discussed and agreed. The framework was then discussed with the wider study team (ND, EW, KM). The interviews that were not transcribed were coded using the agreed coding framework using the detailed notes taken by the researchers during the interviews. New codes were added as they were identified from the remaining transcripts. Codes were grouped together to create themes, through several discussions among all authors. Themes that appeared to be unique to specific timepoints during the pandemic were noted, from the start of the first lockdown in England, to approximately 3 months afterwards.

## Results

### Themes

An overarching theme of uncertainty and reactivity during a crisis was identified from the data. Within this theme, five main themes were identified: concerns about care transitions, uncertainty in engaging support and help, pandemic-motivated care planning, maintaining the wellbeing of the person living with dementia, and trust, loss of agency and confusion.

#### Concerns about care transitions

A common concern that was expressed by family carers throughout the pandemic was whether to make the decision to move the person living with dementia into a care home. When seeking support from support line staff, family carers expressed many concerns about the consequences of admission to care homes, including the lack of visiting opportunities, the potential deterioration of the individual’s condition in the family carer’s absence, and worries about contracting COVID-19 in care homes:

“Lots of worries about no admission to care homes, not being able to visit care homes. What if there's a COVID-19 outbreak in the care home?” (Participant 03)

Requiring to be isolated for a period of 14 days (as per government guidelines) upon admission to a care home was distressing for many people living with dementia, which itself caused worry and upset for family carers:

“If someone goes into hospital, when they go back into the care home they have to isolate in their room for 14 days. As you can imagine, the impact on a person living with dementia and the concern from their relatives is huge.”(Participant 03)

Care transitions between hospital and usual place of residence was found to be a concern for family carers. Support line staff received reports of a lack of communication between staff and family carers, and family carers were not involved with post-discharge decision-making, which often resulted in anger and frustration:

“I think there was a real difficulty about discharge from hospital. And some really complicated and really angry and upset frustrated people when their loved ones were discharged from hospital into care homes. You know that, that choice was made for them.” (Participant 02)

Where family carers had to make decisions about place of care for their relative, support line staff reflected that the decision to stay at home or go to hospital were often made based on fear and worry rather than what carers would actually have wanted:

“I had a call – it was the patient’s wife and she’d had a phone conversation to choose the place of care if he got COVID-19 and he wanted to stay at home, but the wife and son lived at home and they were scared of getting it too if he wasn’t sent to hospital. So these decisions are based on fear and worry rather than preference as such.” (Participant 06)

This fear extended to paid care workers at home too. Many agencies stopped home care visits and family carers took on increased caring responsibilities. To keep the person living with dementia safe, some family carers reported making the decision to move the person living with dementia into their family home rather than, for example, moving them into a care home or having paid carers visiting at home:

“I was having more calls from daughters, or sons occasionally, who had actually moved their parents into their own home, feeling that they did that in the best interest of their loved one, thinking that was the only choice that they had really at the beginning of COVID-19.” (Participant 02)

The above concerns caused carers to feel worse about already difficult decisions (for example, the person living with dementia moving to a care home, then the carer being no longer able to visit them). Carers often reported that it had been hard not being able to visit relatives, and expressed feelings of guilt and feeling under pressure to look after the person:

“There's been a majority of people that are really struggling with that separation. Not being able to visit their loved one, not being able to see them, the same as everybody, really, what we're all going through. But you know, also, being separated from someone that you know is vulnerable and who benefits from that input of seeing you every day.” (Participant 02)

Some carers chose to keep the person living with dementia at home with them at the end of life so that they can ensure that they will be there with them and give them the support they need. Support line staff said that end of life and place of death decisions are difficult even in normal circumstances, but became more of a concern if the person living with dementia had to be moved from one place to another for care due to the pandemic:

“I think concerns about people going to hospital. Especially at the beginning where people were concerned relatives were going to just have to die alone or with staff, not with anyone who loves them.” (Participant 07)

#### Uncertainty in engaging support and help

One of the main concerns reported was accessing health and social care services; particularly getting general practitioner (GP) appointments and accessing support from community nurses. As a result, support line staff reflected that family carers experienced difficulty in building or maintaining resilience due to normal supports not being available, and the increased need to provide more informal care for the person living with dementia.

“GPs aren't available. Memory services aren't available. A lot of Admiral Nurses [specialist dementia nurses] were redeployed into other areas and the service they could offer was changed. It was almost like everything was cut off. Day centres were closed and you know all the support networks that people had in place which is taken away in one fell swoop.” (Participant 04)

Family carers had to decide whether to access healthcare services or continue trying to manage the care needs of the person living with dementia at home without support. For example, some carers were reluctant to contact the National Health Service (NHS) 111 telephone line or call an ambulance for the person due to the pandemic, when they would have done so without hesitation previously. This was due to fears about relatives being taken to hospital, and also about adding pressure to NHS services:

“Many people felt they couldn't call [NHS] 111 if they needed to because of the pandemic […] I think support is there, but in a different way, and that's not always easy and accessible for people.” (Participant 03)

Family carers feelings of unease and uncertainty about calling for help from NHS 111 and emergency services also manifested in other expressions of help:

“I think people have probably been less likely to ask for admissions into hospital for their loved one because of everything else that was going on in the hospital. […] There are a lot of people whose loved ones are falling a lot or having TIAs [transient ischaemic attacks], that they would normally have called 999 [emergency services] and got an ambulance to their loved one, but they'd be less likely so they're struggling through things like that. There's probably a lot of people that needed respite care, but they wouldn't have wanted their loved one to go into respite care, so they've absolutely struggled through this time, not wanting them to go into a care home.” (Participant 02)

This lack of engagement with services was also reflected with family carers expressing feelings of pressure and isolation to helpline staff when making decisions without the support of a health care professional:

“Not being able to do another check with the doctor […] so they weren't able to just have that chat or that support or just supported to make a decision. So I think quite a lot of people were having to make decisions on their own.” (Participant 02)

Staff also reported that family carers became more willing to seek alternative methods of communication and ways of receiving support, this included email support, which was much less common before the pandemic:

“We get a whole host of different callers. But now we have a growing contact base of people who will contact us by email. So we have a lot of emails that we respond to, but they’re always responded to by a nurse.” (Participant 01)

#### Pandemic-motivated care planning

The pandemic highlighted the importance of advance care planning and planning for the future for many carers calling the support line. Carers were focussed on how to make sure that there were provisions in place for the person living with dementia and planning for changes in care that may happen due to the pandemic.

In particular, carers were concerned about making arrangements if they themselves became unwell with COVID-19 and could no longer look after the person living with dementia:

“People felt almost like the doors have been shut on them by every service, and the fear that went with that and what the virus itself meant. What if I become ill and can't look after my relative?” (Participant 08)

Carers had also been encouraged by healthcare professionals to complete Do Not Attempt Cardiopulmonary Resuscitation (DNACPR – also colloquially known as DNR) orders for the person living with dementia. However, support line staff raised concerns that this had not always been conducted sensitively. Some carers contacted support line staff after receiving DNACPR forms in the post to sign without prior discussion with a healthcare professional:

“Initially some decisions were very upsetting for people. Some relatives actually had DNR forms sent through the post, being asked to sign it by a manager of a care home in case this person got COVID, which I was quite horrified by and even telling you now, just the disbelief that was felt.” (Participant 08)

When the person living with dementia was at home and towards the end of life, support line staff were sometimes needed to help family carers prepare for end of life, in particular if they had chosen not to have palliative care teams involved due to the pandemic. This included any plans made, or needing to be put into place by carers:

“The conversation that they might not have too long left to live and haven't got palliative care involved […]. Talking to the person and asking about what they know already, what they've been told already about the person's condition, and if any plans have been put in place […]. That they might need another service brought in, say, spiritual teams. Those kinds of things, with traditional things that need to be written into the care plan.” (Participant 08)

#### Maintaining the wellbeing of the person living with dementia

Many carers had to decide whether to travel to visit the person living with dementia at home and whether to continue to travel in order to provide care and support for them at home. This included worries about the person living with dementia’s condition progressing without adequate, consistent support:

“Lots of concern, anxiety, fear that they would do the wrong thing and endanger their relative, either by offering care or not.” (Participant 07)

“There was a real concern about not only care, but also what impact [not visiting] might have on the cognitive state of someone with dementia - will they remember the person who usually visits?” (Participant 07)

There were difficulties in convincing the person living with dementia to stay at home during lockdown, with many becoming confused and agitated with the restrictions imposed on them:

“A lot of the advice that we're giving is really about how to give reassurance to the person living with dementia because they had become distressed for whatever reason that they can't go out. And people get worried when they see this.” (Participant 03)

Family relationships and dynamics also became more difficult during this time:

“I'm talking about family relationships. I think some families have had to make the decision of which sibling visits their parents, because at one time only one could, if they really had to. So, families were having to make that choice. Family dynamics can be quite difficult and tricky around that subject. But not being able to see their loved ones and be part of their lives at the moment and just totally trusting them to someone else, I think it’s hard.” (Participant 04)

During the later stages of the first lockdown in the UK, carers expressed concerns about having to return to work and therefore being less available to provide care:

“Issues around people now going back to work after being at home and caring for somebody and that this persons now got used to them being at home.” (Participant 03)

Other concerns included family carers experiencing challenges when shopping for food and necessities for the person living with dementia:

“Even down to online shopping, priority slots weren’t given to carers who have somebody living with dementia, despite the fact they can't leave them to go out shopping on their own because supermarkets only allow one person at a time.” (Participant 03)

#### Trust and confusion

Issues of trust, perceived loss of agency and confusion regarding government guidelines were expressed throughout the first-wave of the pandemic.

There was reduced reliance on healthcare professionals, which was sometimes due to a lack of availability of staff such as community nurses, or because of a lack of communication - for example, hospital staff deciding on discharge plans without consulting family carers. This lack of trust resulted in an increased use of alternative sources of support and advice, such as charity helplines. Support line staff said that family carers found it helpful when nurses answered calls, as they were perceived as trustworthy and reliable due to being qualified, regulated professionals:

“I think there's something about contacting a specialist nurse who they perceive will understand because they work with people with dementia, and for many of the families, that's been the comment; ‘I can't get hold of my doctor, we’re not involved with other professionals, there's no services out there’. So they're not just seeking an answer from someone on the end of the phone, there is definitely something about them contacting a nurse who is a health professional […], someone who is a registered nurse, just as they would with contacting the doctor or other health professionals.” (Participant 01)

Government guidelines were a major source of concern for family carers, as they frequently changed and were often unclear. This led to carers feeling forgotten or left out, and that they couldn’t trust the government to consider the needs of person living with dementia and their carers. Family carers worried about getting into trouble with the police if they were ‘caught’ doing something they were not sure was allowed, such as travelling to visit a relative.

“Everything that was suddenly happening with no warning, uncertain guidance, sudden announcements or changes to what was told two days ago or whatever. And now what am I going to do because they said this? Two days ago we could do this now, they said that now what? Now what am I allowed to do?” (Participant 08)

## Discussion

This paper explored challenges faced by family carers of people living with dementia and the decisions they had to make during the first wave of the pandemic in England, in particular focussing on those caring for people living with dementia towards the end-of-life. We identified an overarching theme of uncertainty and reactivity during a crisis, and within this, five main themes were identified including: concerns about care transitions; uncertainty in engaging support and help; pandemic-motivated care planning; maintaining the wellbeing of the person living with dementia, and trust, loss of agency and confusion. This is the first paper, to our knowledge, to explore the concerns and help seeking behaviours of carers of people living with dementia during the COVID-19 pandemic, from the perspective of leading UK charities in dementia and end of life care.

There has been a lot of media attention during the pandemic on the deaths of people living with dementia from COVID-19 in the UK, especially with reports of dementia being the most common underlying condition for COVID-19 deaths [1, 2]. However, we found that many people were not calling the helplines specifically in relation to end of life issues but an array of issues which could be challenging throughout the stages of dementia.

The pandemic has highlighted significant gaps in the provision of social care for people living with dementia and their carers. As a result, the Alzheimer’s Society have advocated that “the Government must urgently produce a long-term social care solution with sufficient funding, to protect both people with dementia and their carers” [16].

Notably, trust was a key element which had been affected by the pandemic - including trust in the government and their guidelines. COVID-19 has had a huge impact on both people living with dementia and their carers. In particular there has been the removal of several social support services [3, 17]. Our findings indicate that carers also struggled to obtain support from health care services such as community nursing or Admiral Nurses as these professionals were redeployed to other parts of the health service. This impacted carers’ experience of dependability of services, which we know is important in delivering high quality dementia care [18].

The nature of the pandemic has meant that carers have been hesitant and less likely to seek help in the absence of these services [19], in particular support from NHS services such as 111 or ambulance services where needed. However, our results suggest carers have sought alternative sources of support, and the support line staff from charities interviewed for this study have helped fill the gap, with more carers turning to them than before. The preference for calling a charity over the NHS advice line, as evidenced by callers to the helplines, may indicate an increased need for holistic, personalised support during the pandemic. It could also indicate a perceived sense that charities do noy have the same pressures as the NHS, for example, the message from the media was that the NHS was overwhelmed during the pandemic and carers may have felt it was important not to contribute to this.

Telephone support lines, online communities and other mechanisms including email have been an invaluable source of support for carers during the first wave of the pandemic, as expressed by the callers as well as the support line staff. Support line staff felt that trust in professionals shifted during the first lockdown, when many services were not equipped or prepared to adapt to the rapid changes taking place. However, telephone support lines had dedicated and trained staff familiar with the unique position of being a carer for a person living with dementia. The increased use of these services mirrors what is seen in the current health service with more alternative digital approaches adopted to delivering healthcare and recommendations for maintaining care [20]. Our results are also reflected in reports from the Alzheimer’s Society and their Dementia Connect service which highlighted the lack of availability of support available across services, and the increased strain on family carers [21].

The pandemic has brought to the foreground for many people the prospect of death and dying. This is particularly evident in the calls that participants in this study reported. Participants highlight that motivations for advance care planning was the acknowledgement of the potential carer’s death and mortality, and not always the potential death of the person with dementia. In non-pandemic times, advance care planning is motivated by the prospect of the person with dementia dying and not the carer themselves. Advance care planning has been shown to improve outcomes including burdensome transitions and carer outcomes such as decisional conflict [22]. Despite guidance recommending that discussions about end of life care and advance care planning should occur early with the person living with dementia while they have capacity [23], many do not complete a plan and this has been made apparent during the current pandemic [7]. Despite encouragement during the pandemic to complete advance care plans [24], many may no longer have the capacity to do so. Families are therefore likely to be relied upon by professionals to understand previously expressed preferences and wishes to make a decision about care in the best interests of the individual [25].

However, this study shows how planning and engaging families in significant discussions and decisions during the pandemic were not always undertaken sensitively, in particular DNACPR decisions. Clear, sensitive and ongoing communication with family is important, especially when making decisions not just during the pandemic but also beyond. We know that in non-pandemic times lack of continuity and coherent care pathways can negatively impact decision making [26]. However, this may be heightened during the pandemic, making communication and decision making more complex. Often decisions are not a carer’s decision to make alone, however, within the context of a pandemic they may feel isolated and solely responsible for decisions about their relative’s care. It is vital that carers are supported and do not take on this burden of responsibility. It is important that carers understand support is available and they should not be afraid to seek support, as multidisciplinary professional involvement aids decision making and helps achieve goals of preferred place of care and place of death [27, 28].

The pandemic has changed decision making around place of care. Care at home appears to be the preferred option [27]. This may reduce COVID-19 risk and ensure families can continue to see their relatives, but brings its own challenges and difficulties. These include carers struggling to cope with increased caring responsibilities with limited support from external services [8] and having to source advice and information independently regarding end of life care.

### Implications for research, clinical practice and policy

The reduced availability of services may mean that family carers are not receiving the support they would normally be getting outside of the pandemic. The increase in calls, and carers taking on additional caring responsibilities, also suggest that family carers need further support than normal during the pandemic. In particular, decision making appears particularly challenging, with family carers making more decisions than they would have done previously. We know family carers are currently faced with the prospect of many end of life decisions and these are decisions they find particularly difficult [29]. End of life decisions require strong relationships between health care professionals and carers, however these were fractured during COVID-19 when health care professionals were under enormous pressure to care for an influx of very acutely unwell and dying patients. Professionals find these discussions difficult in non-pandemic times [30], but during the pandemic such discussions are even more difficult, and have been constrained by logistical pressures. There is a need to support carers during the pandemic when support is limited and feelings of isolation in making decisions are strong. While professionals are under enormous pressures there is a need for alternative forms of support.

The research findings from this study were used to inform the development of a decision aid to support family carers of people living with dementia when making end of life care decisions during the pandemic. This decision aid can be directly distributed and downloaded by carers but it also provides a resource for professionals to use when discussing decisions and plans with families. The decision aid has been distributed nationally and is part of NHS England Dementia Wellbeing in the COVID-19 pandemic pathway and can be accessed here: http://www.ucl.ac.uk/psychiatry/decision-aid [31, 32].

### Strengths and limitations

This is the first study to directly interview telephone support line staff who are working on the front line handling calls and messages from family carers experiencing difficulties and concerns during the pandemic. They have given us an insight into the experiences and needs of carers who are potentially in most distress and struggling and therefore unlikely to take part in research studies. Our findings come with the caveat that these are second hand accounts of carer concerns and the decisions they made during the pandemic. However, secondary analysis of non-governmental organisation (NGO) data has been a successful method of facilitating research in previous studies [33].

This is a small sample and therefore transferability may be limited, however given the time period in which these interviews took place (first peak in the UK), recruitment was difficult as staff were exceptionally busy and were dealing with a global pandemic supporting the most vulnerable. It is important to share these findings which have important lessons and implications, and we doubt the results would have been altered considerably with further interviews.

Many of our participants were qualified nurses and therefore may have elicited different responses from family carers on their calls, for example more clinical concerns. However, many topics discussed are wider than simply clinical concerns. Qualified nurses may have increased trust in conversations and encouraged discussions.

## Conclusions

The pandemic has had a detrimental impact on the availability of services and support that family carers and people living with dementia could access. Carers are reluctant to seek support because of fear of what will happen to their relative, a lack of communication between carers and professionals, and not wanting to burden the NHS. Instead, they are seeking support from alternative services they trust, such as nationally known charities.

## Data Availability

The datasets analysed during the current study are available from the corresponding author on reasonable request, and subject to ethics and data protection requirements.

## Abbreviations

DNACPR: Do Not Attempt Cardiopulmonary Resuscitation
GP: General Practitioner
NGO: Non-Governmental Organisation
NHS: National Health Service
PPE: Personal Protective Equipment
TIAs: Transient Ischaemic Attacks
UK: United Kingdom
